# FTIR Spectroscopic Imaging Supports Urine Cytology for Classification of Low- and High-Grade Bladder Carcinoma

**DOI:** 10.3390/cancers13225734

**Published:** 2021-11-16

**Authors:** Monika Kujdowicz, Brygida Mech, Karolina Chrabaszcz, Piotr Chlosta, Krzysztof Okon, Kamilla Malek

**Affiliations:** 1 Department of Pathomorphology, Faculty of Medicine, Jagiellonian University Medical College, Grzegorzecka 16, 31-531 Krakow, Poland; monika.kujdowicz@uj.edu.pl; 2 Faculty of Chemistry, Jagiellonian University in Krakow, Gronostajowa 2, 30-387 Krakow, Poland; brygida.mech@student.uj.edu.pl (B.M.); karolina.chrabaszcz@ifj.edu.pl (K.C.); 3 Department of Experimental Physics of Complex Systems, Institute of Nuclear Physics, Polish Academy of Sciences, Radzikowskiego 152, 31-342 Krakow, Poland; 4 Department of Urology, Faculty of Medicine, Jagiellonian University Medical College, Jakubowskiego 2, 30-688 Krakow, Poland; piotr.chlosta@uj.edu.pl

**Keywords:** bladder carcinoma, infrared spectroscopic imaging, diagnostics, cytology

## Abstract

**Simple Summary:**

Human urine cytological samples were investigated using Fourier transform infrared spectroscopic imaging in terms of recognition of bladder cancer. The clustering of IR spectra of whole cytological smears revealed very good spectral correlation with normal urothelial cell features. Next, the combination of spectral information derived from unsupervised hierarchical cluster analysis and partial least square discriminant analysis (PLS-DA) classified normal vs. low- and high-grade bladder urothelial carcinoma with sensitivity and specificity of 90–97%.

**Abstract:**

Bladder urothelial carcinoma (BC) is a common, recurrent, life-threatening, and unpredictable disease which is difficult to diagnose. These features make it one of the costliest malignancies. Although many possible diagnostic methods are available, molecular heterogeneity and difficulties in cytological or histological examination induce an urgent need to improve diagnostic techniques. Herein, we applied Fourier transform infrared spectroscopy in imaging mode (FTIR) to investigate patients’ cytology samples assigned to normal (N), low-grade (LG) and high-grade (HG) BC. With unsupervised hierarchical cluster analysis (UHCA) and hematoxylin-eosin (HE) staining, we observed a correlation between N cell types and morphology. High-glycogen superficial (umbrella) and low-glycogen piriform urothelial cells, both with normal morphology, were observed. Based on the spectra derived from UHCA, principal component analysis (PCA) and partial least squares discriminant analysis (PLS-DA) were performed, indicating a variation of protein content between the patient groups. Moreover, BC spectral cytology identified a low number of high-glycogen cells for which a shift of the carbohydrate/phosphate bands was also observed. Despite high cellular heterogeneity, PLS-DA was able to classify the spectra obtained. The voided urine FTIR cytology is one of the options that might be helpful in BC diagnosis, as high sensitivity and specificity up to 97% were determined.

## 1. Introduction

Bladder urothelial carcinoma (BC) cytological diagnosis awkwardness is broadly discussed in the literature [1,2]. This commonly used diagnostic method is based on examination of many subjective morphological features of cells present in urine, which, according to the diagnostic standard, are usually stained with hematoxylin and eosin (HE). Cytological sensitivity for LG BC is c.a. 30–50%, whereas for HG BC, it is 80% [1,3]. The reason for such a difference in sensitivity is the fact that a part of LG BC cells might have similar features to normal cells or show no morphological changes. Thus, diagnosis is put forward after tissue excision and the occurrence of a thickened urothelium with disrupted stratification. In spite of this diagnostic pitfall, LG BC rarely infiltrates, and often creates papillary structures which are easily visible in cystoscopy and ultrasound [1,3,4,5]. If BC is suspected in cytology, both quantitative (over 5 cancer cells) and qualitative criteria should be observed. The features of BC include: high nuclear/cytoplasmic ratio and nuclear diameter, hyperchromasia and irregular shape of nuclei, chromatin coarseness, and the presence of inclusions, nucleoli and atypical mitoses [6]. Detailed morphological features and grouping according to the Paris system are presented in Table S1 in Supplementary Materials (SM). BC cytology is cumbersome and subjective, but apart from this, it is cost-effective and satisfactory in the detection of highly malignant HG BC [6]. Furthermore, the BC subtypes have different biocomponent contents, but according to their occurrence, molecular pathways and behavior, the dichotomic classification to LG and HG BC underlines the most important differences in BC [3,7,8]. The whole cytology sample contains hundreds of cells that need to be laboriously examined by a pathologist, and the main aim during urine cytology testing is to identify BC cells.

Due to functional differences between cells exfoliated to urine from different urothelial layers, the morphology and biochemical composition can vary [3,9]. Urothelial cells have different abilities to change their diameter when fulfilling the bladder or during urination, in addition to varying proliferation rates. Moreover, urothelial cells are differentially exposed to metabolites, infection factors and distance to vasculature within the subepithelial tissue, which in turn relates to oxygen and nutrition accessibility [3,10]. New tools for the detection of bladder cancer cells are offered by Fourier transform infrared (FTIR) and Raman spectroscopies with imaging modality. Any biological object is identified by a specific set of bands in the spectra assigned to vibrations of proteins, lipids, nucleic acids, and carbohydrates. The complex molecular signature in both spectra undergoes changes upon stress conditions and pathological processes. However, no patients’ cytological cells have yet been imaged with detection through FTIR and Raman spectra. The only urothelial cell imaging to date was performed on cell cultures [11,12]. A single-point microscopic examination of urothelial cells in urine from healthy volunteers showed the differentiation of three cell types only [10]. Furthermore, classification of urine sediment to normal or BC groups were based on the attenuated total reflectance (ATR) FTIR spectra of bulk samples [9,13,14,15]. Complementary to the FTIR method, Raman spectroscopy also showed promising outcomes for the spectroscopic scanning of malignant and benign bladder tissues [16]. These few studies showed diagnostic accuracy of over 80%.

This study was performed with the aim of assessing whether label-free infrared spectroscopic imaging of urine cytology can help to classify normal, LG and HG BC samples. We proposed here a novel approach for data collection and analysis to extract relevant information for patient classification despite the heterogeneity of urothelial cells and their transformation due to malignancy. Our spectral approach was verified by clinically used cytology.

## 2. Materials and Methods

The study was reviewed and accepted by the First Local Ethical Committee at the Jagiellonian University Medical College in Krakow (No. 1072.6120.100.2018). Urine samples from 45 patients with clinical suspicion of BC were collected. The inclusion criteria included clinical suspicion of tumor and age over 18 years. We excluded pregnant women, patients with infection and after radiotherapy, and samples of very low cellularity. Firstly, each fresh (up to 30 min after urination) whole voided urine sample was centrifuged at 2000 RPM for 5 min and divided to perform standard cytology and FTIR spectroscopic imaging. For the first method, urine samples were spread with cytospin (Thermo Scientific Cytospin 4) and fixed with 95% ethanol. The urine standard diagnostic cytology cytospin slide is a 7 mm diameter circle (ca. 38 μm^2^) filled with unevenly distributed cells. Next, the samples were HE stained and assessed according to the Paris System for Reporting Urinary Cytology [6]. Based on this examination, samples were assigned to three groups with normal, low- and high-grade bladder cancer cells (N, LG, and HG BC, respectively). Each group consisted of 15 patients. Examination and photographic documentation were performed using an Olympus BX53 white-light microscope equipped with an Olympus DP27 digital camera (Department of Pathomorphology, University Hospital, Krakow). 

The remaining fresh urine sediment was spread with the cytospin on a CaF_2_ window, and afterwards fixed with 2% glutaraldehyde (30 min), washed with distilled water and dried (min. 48 h in a desiccator). A sample of 7 μm diameter was imaged with standard definition FTIR spectroscopy in transmission mode. To scan the entire area of the sample, 3 mosaics with an area of 2100 × 2100 μm were acquired. For this purpose, a FTIR Agilent 670 spectrometer was employed which was equipped with a 128 × 128 FPA camera with a pixel size of 5.5 × 5.5 μm and Cassegrain objectives (NA = 0.62). FTIR spectra were acquired in the region of 900–3700 cm^−1^. A total of 64 scans were co-added with a spectral resolution of 8 cm^−1^. Afterwards, the samples were stained with HE for direct comparison of the IR images with cell morphology.

Pre-processing and chemometric analyses were performed using CytoSpec (ver. 2.00.01), MatLab (R2020a, MathWorks, Natick, MA, USA), Unscrambler X (v. 10.5.1, Camo, Montclair, NJ, USA), OPUS (ver. 7.2.139.1294, Bruker, Billerica, MA, USA) and Origin 9.1 (ver. 2020b, OriginLab program, OriginLab Corporation, Northampton, MA, USA) software. Water vapor correction, Q-tests and PCA denoising were performed (Cytospec and Matlab) as in Kujdowicz et al. [12]. The Q test excluded IR pixel spectra with low signal-to-noise ratio (SNR) which could lead to false analysis. As reference of a high SNR spectrum we used maximum value of absorbance gathered in the 1700–1600 cm^−1^ region while noise was calculated as the standard deviation of spectra in the 1900–1800 cm^−1^ region. These steps allowed us to reduce a number of spectra to ca. 0.5 million per sample and next to apply a 6-class unsupervised hierarchical cluster analysis in the 3000–2820 and 1780–980 cm^−1^ regions (UHCA). The FTIR spectra derived from the UHCA were preprocessed similarly as in Gajjar et al. [17]. The UHCA mean spectra were cut below 950 cm^−1^, and Rubberband baseline and vector normalization (1780–1000 cm^−1^) were performed (OPUS). PCA was performed on 810 derivative spectra derived from UHCA (270 spectra per patient group) in the regions of 3000–2820 and 1780–975 cm^−1^ with a NIPALS algorithm, and mean-centered data, identifying outliers and maximum 7 components. To create the PLS-DA model, firstly a Ward distance cluster analysis (CA) was performed on 18 UHCA-derived mean spectra to extract nine spectra for each patient. The remaining nine spectra were left for prediction (Unscrambler). CA and PLS-DA were performed in the same spectral region as the PCA. In the PLS-DA models, four factors were used with mean centered data. The integration intensities of the amide I (1690–1630 cm^−1^) and glycogen (1180–1120 cm^−1^) bands were calculated from second derivative spectra (OPUS). All graphs were depicted using Origin software.

## 3. Results

### 3.1. Clinic-Pathological Profile of Patients

According to standard HE cytological examination of voided urine, 45 patients were grouped into three groups with normal cytology (N), low-grade BC (LG BC) and high-grade BC (HG BC). Each group included 15 patients. Table 1 summarizes the clinical data of patients, including gender, age, hematuria and urine pH.

### 3.2. Cluster and Principal Component Analysis of Spectral Database 

**Figure 1:**
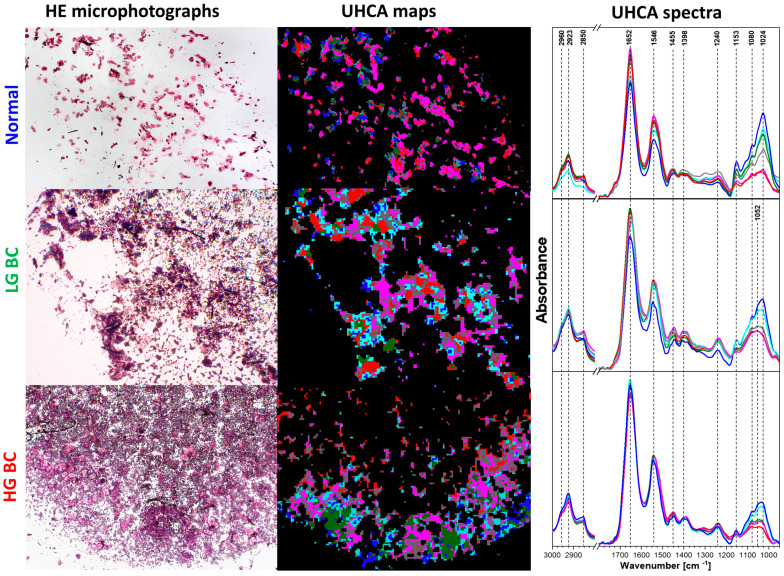
Comparison of the HE microphotographs of normal, LG BC and HG BC cytology (magnification: 100× for normal and LG BC samples and 50× for HG BC) with false-color UHCA maps from IR images and their mean absorbance spectra. The colors of the spectra correspond to the colors of UHCA classes.

The first step of image preprocessing was water vapor removal and the Q-test, which allowed us to reveal pixels with high signal-to-noise FTIR spectra from the area covered by large urothelial and squamous cells (>10 μm in diameter). Small cells, like lymphocytes and erythrocytes, were too thin. Their spectra showed a substantial scattering effect, and they were rejected from further analysis. Cytospin deposition of cells on an IR-transparent substrate caused an accumulation of the large cells on the periphery of the deposit. Cellularity varied across patients and it did not depend on the group. According to the literature, urine sample cellularity strongly depends on desquamation, urine volume, time between urinations and the physical activity of patients [3,6,10]. A clear morphological-spectral assignment was achieved only for four cell classes from healthy patients (N group), i.e., glycogen-rich cells, including urothelial superficial (umbrella) and squamous cells (a clear morphological distinction between the two is impossible); umbrella glycogen-poor cells, and piriform and basal cells from the deep layers of the bladder (Figure S1 in SM). Classes of unsupervised hierarchical clustering (UHCA) were assigned to these cells based on their HE morphology. This example could suggest a straightforward classification of the urothelial and cancer cells, but we abandoned that approach because it was impossible to select a proper number of UHCA classes with a high agreement with cell types, in particular LG BC cells. Therefore, our approach for further image analysis involved obtaining the six UHCA blind spectral classes. This number of classes was a compromise between the expected cell types and the observed spectral variability in the IR spectra. This idea of urothelial FTIR-based cytology is presented in Figure 1. 

We analyzed IR spectra in two regions: the high-wavenumber region (3000–2800 cm^−1^) assigned predominantly to lipids and proteins (vibrations of the CH_3_ and CH_2_ groups), and the fingerprint region (1800–1000 cm^−1^). Usually, the most intensive bands in the fingerprint region belong to proteins (1700–1500 cm^−1^) and carbohydrates and moieties with the phosphate group (1200–1000 cm^−1^) [18,19]. Detailed assignments of IR bands to biomolecules are shown in Table S2 in SM [20,21,22,23,24,25].

The largest spectral differences between the six UHCA classes from one image in all samples were found in the bands assigned to proteins (amide I—1652 and amide II—1548 cm^−1^) and in the carbohydrate-phosphate region (1200–1000 cm^−1^), particularly in the bands at 1153, 1070 and 1024 cm^−1^ assigned to glycogen and/or glycolipids constituting urothelial cerebrosides [20,21,22]. The comparison of HE microphotographs and UHCA maps indicated that the high-glycogen classes present in all patient groups (light blue and blue traces in Figure 1) originated from large and superficial normal cells with a low nuclear-cytoplasmic ratio (umbrella and squamous cells). The low-glycogen classes (pink, red and green traces in Figure 1) presented thick eosinophilic-type umbrella, piriform, basal and cancer cells. And all of these low-glycogen cells exhibited high nuclear-cytoplasmic ratios. These observations were congruent with our previous spectroscopic studies on urine sediments and the reference cell cultures of urothelial and BC cells [11,14]. Spectral differences between the N and BC groups were seen the shapes of the glycogen bands, e.g., a sharp 1024 cm^−1^ band was present in N while an additional 1054 cm^−1^ band was found in both BC groups. A visual inspection of the mean FTIR spectra also suggested a higher content of carbohydrates in LG BC cells than in HG BC (Figure 1). 

**Figure 2:**
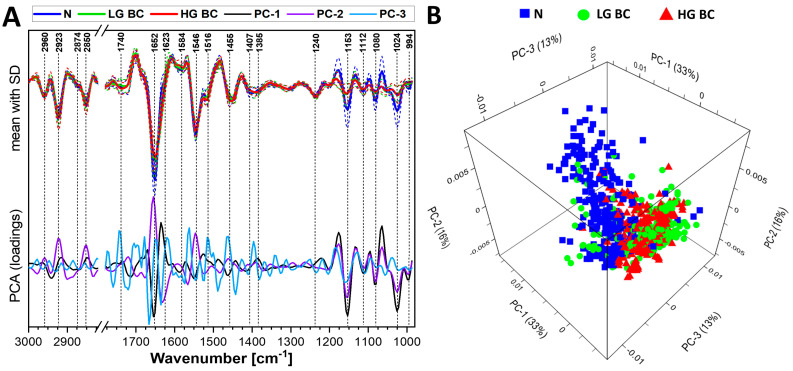
(**A**). Averaged second derivative FTIR spectra (±SD—dashed lines) and PC 1-3 loadings plots from principal component analysis (PCA). (**B**). 3-dimensional PCA scores plot. Averaged spectra and PCA were calculated from 270 spectra for each patient group (810 in total).

Spectral intragroup variability and intergroup differences between the N, LG and HG BC groups were presented in averaged FTIR spectra (Figure 2A and Figure S2 in SM) as well as in the PCA loadings and score plots (Figure 2 and Figure S3 in SM). The normalized averaged spectrum should be interpreted as the relative amount of major biocomponents and the vibrational activity of the chemical groups, crystal structure and environment near atoms [23,24,25,26]. The carbohydrate-phosphate region showed high intragroup variability, especially in spectra derived from urine of healthy patients. Undoubtedly, this was a reason for the recognition of specific urine cells, see Figure S1 in SM. In neoplasm cases, the clonality of cells and the appearance of neoplastic changes in the whole bladder urothelium (not only in tumors) explain the low intragroup variability in the investigated BC groups. 

A total of 810 derivative FTIR spectra obtained from UHCA analysis of IR images were used to perform the principal component analysis (PCA), which is an unsupervised method for grouping spectral data; here N, LG BC, and HG BC. All of the groups were segregated along the main principal components (PC-1—PC-3) with a total variance of 62% (Figure 2A). The score plots of these PCAs are summarized in Figure 2B and Figure S3 in SM. The PC-1 axis grouped FTIR spectra of the N (positive scores) and BC groups (negative scores) accorded to discriminators attributed to a glycogen level (1153, 1080 and 1024 cm^−1^) and the height and shape of the amide I band at 1652 cm^−1^. The PC-2 axis, similarly to PC-1, indicated the contribution of the glycogen bands and the red-shift of amide I band to the observed grouping. Here, we also found positive vectors at 2850 and 2923 cm^−1^ assigned to stretches of the CH_2_ group in the long-chain fatty acids. The majority of the LG BC spectra were clustered along the negative PC-2 axis, contrary to HG BC. Furthermore, the PC-3 loadings and scores plots which indicated alternations in protein conformations (discriminators at 1652 and 1623 cm^−1^) showed both groupings in the BC patients. 

**Figure 3:**
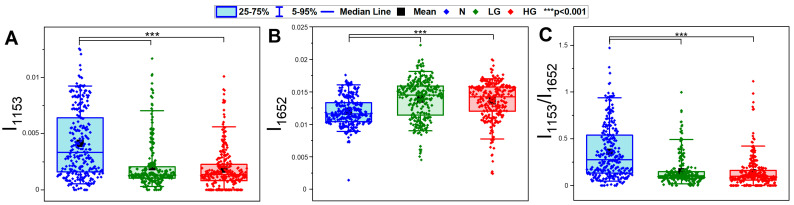
The integration of: (**A**) glycogen (1153 cm^−1^), (**B**) proteins (1652 cm^−1^), and (**C**) carbohydrate/protein ratio. Integral intensities were calculated from second-derivative FTIR spectra; 270 spectra per each group (810 in total).

Since the glycogen and protein bands contributed to PCA discrimination, the integral intensities of these bands were calculated next. The integral intensity of the 1153 cm^−1^ band assigned predominantly to glycogen was higher in the N group than it was in both BC groups, reverse to protein integral intensity and similarly to the carbohydrate/protein ratio (Figure 3A–C, respectively). The opposite trend was determined for the protein level (Figure 2A). The distribution of intensity values showed that the content of glycogen significantly varied across healthy patients, in contrast to both BC groups. Here, there was a population of cells exhibiting high intensity in the 1153 cm^−1^ band, and this fact likely resulted from the presence of healthy urothelial cells among the malignant ones. Such a situation was not found for the content of proteins.

### 3.3. PLS Discrimination Analysis of Patients’ Groups

**Figure 4:**
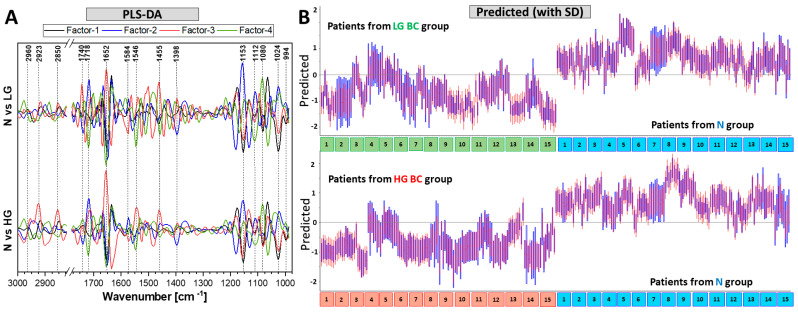
(**A**) PLS-DA loadings (N with positive scores). (**B**) Predicted values, calculated in pairs of N vs. LG BC and N vs. HG BC (red—SD, blue—unexplained by model variance). A total of 135 derivative FTIR spectra from each group (9 spectra from each patient) were taken to build the model, and the remaining 135 spectra were used for prediction. The spectra above zero are predicted as N, and spectra below zero are predicted to be LG or HG BC, respectively.

**Figure 5:**
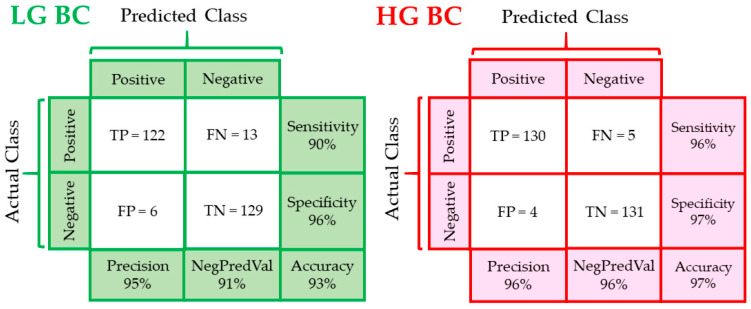
Confusion matrices for N vs. LG BC groups and N vs. HG BC, calculated from PLS-DA results. Abbreviations: TP—true positive, FN—false negative, FP—false positive, TN—true negative, NegPredVal—negative predictive value.

A four-factor PLS-DA classification based on second-derivative FTIR spectra was performed in pairs of N vs. LG and N vs. HG. We also examined the LG and HG BC groups; however, seven factors were needed to obtain satisfactory classification, and we excluded this method for its low-robustness. In the first step of the PLS DA analysis, we divided the spectra into two groups to build the model, and then to test its prediction ability. The model groups were selected according to a strategy proposed by Lee and co-workers [26]. A nine-class hierarchical cluster analysis (HCA) was performed to select the data set for modelling and prediction, as seen in the exemplary HCA diagrams in Figure S4 in SM. This was performed on 18 UHCA spectra derived from IR images separately for each of 45 patients. The nine IR spectra with the largest spectral variability were taken to construct the PLS-DA model. These results are summarized in Figure 4 and Figure 5.

The PLS loading plots showed similar molecular differences between the groups as the PCA loadings (Figure 2A and Figure 4A). Detailed PLS-DA parameters are presented in Table S3 in SM. The sensitivity and specificity of LG BC discrimination from healthy patients were 90 and 96%, respectively. The corresponding values of 96 and 97%, respectively, were determined for HG BC vs. N (Figure 5). 

In each PLS-DA model, 135 spectra were taken from each group to build the model and 135 spectra were used for prediction. The prediction results and the HE assignment of patients to N, LG and HG BC were used to construct the confusion matrices (Figure 5). Six and four IR spectra from the normal group were false-positively (FP) assigned to LG and HG BC, respectively. In turn, thirteen LG BC and six HG BC spectra were classified as normal cytology (false negative, FN). The accuracy was much better for HG than LG BC (97% vs. 93%). The precision values were 95% and 96%, while the negative predictive values were 91 and 96% for LG and HG BC, respectively. 

## 4. Discussion

The main differences between the N and BC groups were found in protein and carbohydrate regions. A part of normal cells with the low-glycogen bands had similar scores in the PCA as those in LG and HG BC groups. These similarities resulted from the biological nature of proliferating cells, i.e., cells that proliferate quickly, have a low cytoplasm to nuclear ratio and have cytoplasm can contain spare substances. These differences between nuclei and cytoplasm were discussed in a report showing the IR features of urinary cell cultures detected by high-definition FTIR imaging [11]. Here, scanning of cytological samples with a focal plane array of a 5.5 × 5.5 μm pixel size did not allow for the observation of carbohydrates in the cell compartments. Our current approach to discriminate cells spectrally according to their morphological type was fully successful in the N group only, similarly to Bird et al. [9] Therefore, we assumed that the increased intensity of the glycogen/glycolipid bands could have been related to the function of some urothelial or squamous cells. The distribution of intensity of the 1153 cm^−1^ band in the N group suggested three spectroscopic cell types, whereas the mean and median values of this spectral parameter were similar for both BC groups (Figure 3A). The 1200–1000 cm^−1^ region of the normal morphology cells in the BC sample was not only less intense but also its shape changed. The IR spectrum showed the presence of an additional band at 1052 cm^−1^ assigned to DNA, cholesterol or glycolipids, and flattened bands at 1080 and 1024 cm^−1^ assigned to glycogen (Figure 1). 

The PLS-DA models were similarly efficient in classifying healthy patients from those with diagnosed low- and high-grade bladder cancer (Figure 4 and Figure 5). A high spectral variability in HG BC did not allow the building of a model discriminating both BC groups, as indicated by the PC-2 and PC-3 scores shown in Figure 2A. Single FTIR spectra from different N patients were false-positively subjected to LG and HG BC (six and four spectra, respectively). These spectra resembled the IR signatures of BC urothelial cells, but other spectra for a given patient were classified correctly. This suggests that large-area FTIR scanning of the 30 μm^2^ smear of cells isolated from urine was efficient to recognize healthy patients. In the case of false negative results, only one LG BC patient was misclassified based on majority of the IR spectra, whereas two LG BC and three HG BC cases were assigned to the healthy group according to single spectra only. Such doubtful assignments can be further verified by classic cytology and histology. Our HCA-PLS-DA models of the imaged cells showed better sensitivity and specificity (both above 90%) than other FTIR-based classifications performed on bulk samples of patients’ urine sediment (sensitivity: 100%, specificity: 59%) and urine (sensitivity: 90%, specificity: 81%) [14]. The high PLS-DA values of accuracy and other classification indicators determined in this work are more optimistic, and some factor of the proposed approach played an important role for achieving these results (Figure 5). 

It is worth highlighting the great potential of the large-area scanning by FTIR spectroscopic imaging of all cells in the sample, the removal of numerous small cells (lymphocytes, erythrocytes, basal and cytolytic cells as well as debris) from the analyzed IR images and the segregation of spectra with different glycogen and protein contents. FTIR imaging of cells is much faster than Raman mapping due to detection by the focal plane array incorporated in the IR microscopes that covers a large area of the sample in single measurement. This technique was employed to investigate biopsy samples as well [7], but FTIR-based cytology offers non-invasive sample collection from patients. We showed in the previous work that simple ATR FTIR spectra of the whole urine sediment can be also used for the recognition of BC. However, this method showed lower sensitivity and specificity than the approach proposed here due to averaged IR signatures and the hidden spectral features of single cancer and abnormal cells in such a bulb sample [14]. Here, we detected a spectral variation among urine cells that was particularly important in the case of the samples with low fractions of BC cells typical of the early stages of the disease and small tumors [27,28]. 

We also concluded that the differences in sensitivity and specificity between the patient groups resulted from different biochemical features of N and BC urothelial cells located in their compartments. LG BC cells predominantly grow, slowly and are often exfoliated from papillary hyperplasia in the early, non-invasive stages, whereas HG BC cells infiltrate very quickly from the beginning of the malignant process and cause degradation of bladder wall matrix and detritus in urine [8]. Therefore, classification based on the spectra of single cells is more accurate than classification based on urine and urine sediment containing various cell types and matrix mixtures. A similar observation was found for the FTIR-based classification of cervical cancer and the spectral Raman cytology of cancer [16,27,28,29,30,31]. This is also an important conclusion in terms of the verification of ambiguous cytological results by biopsy and histopathological examination. The bladder tissue is composed of a plethora of cell types (e.g., epithelial cells, fibroblasts, myocytes, and lymphocytes) and a tissue matrix, and thus pathological changes are mainly affected by local changes of the epithelium as well as general patient conditions. All of these features made the histological classification difficult. 

To sum up our discussion, the results showed the reliability of FTIR spectroscopic imaging for the recognition of both grades of bladder cancer. Further development of the automized analysis could serve as a first screening diagnostic tool in clinics, especially for the identification of the LG BC cases difficult to diagnose by cytology. This approach has great potential to support histopathological examination since this method is label-free and a typical experiment with this analysis can be completed in a few hours. This method could be further used for the fast tracking of the disease progression and personalized treatment.

## 5. Conclusions

In this work, we performed FTIR spectroscopic imaging of whole cytology samples prepared from voided urine cytology to recognize their biochemical diversity across hundreds of cells. The highest intragroup variability with the presence of three spectral cell types was demonstrated for the N group. The most significant changes between the N and BC groups were found in the carbohydrate and protein region, whereas the lipid vibrational modes indicated differences between HG and LG BC. After the CA-based selection of IR spectra to build the PLS-DA model, we achieved high sensitivity and specificity for the discrimination of both BC groups from healthy patients (90–96% and 96–97% for LG and HG BC, respectively). Spectral urine cell cytology could potentially be implemented as a screening test with non-invasive collection of diagnostic samples, simple preparation, and label-free and automatic sample classification. 

## Figures and Tables

**Table 1:** Clinical profile of the investigated patients.

	N	LG BC	HG BC
Gender [M/F]	3/12	8/7	14/1
Age [ys ± SD]	64.9 (13.7)	70.8 (5.6)	70.9 (6.0)
Hematuria	1	3	0
Urine pH [±SD]	5.57 (0.56)	5.57 (0.65)	5.57 (0.56)

## Data Availability

A hard drive in the Department of Pathology CM UJ.

## Supplementary Materials

The following are available online at https://www.mdpi.com/article/10.3390/cancers13225734/s1, Figure S1: The comparison of HE staining and UHCA-discriminated cells of normal urothelial cells showing their assignment; on right, mean FTIR spectra of UHCA classes (the colors of the spectra correspond to the colors of classes in the UHCA map), Figure S2: **A.** Averaged absorbance FTIR spectra (with SD marked as dashed lines). **B.** Median, 1st and 3rd quantile and min and max plots. All spectra were preprocessed after baseline correction and vector normalization, Figure S3: 3D PCA scores plots from Figure 2B showed in different projections, Figure S4: Example of cluster analysis from one of N, LG and HG BC patients. Spectra from the most different branches of Cluster Analysis were chosen to build the model, were marked with arrows and they represent 9 out of 18 spectra from the patient, Table S1: Normal, LG and HG BC cell features according to the Paris system, Table S2: Positions of IR bands observed in FTIR spectra of urine sediment with their assignment to vibrational modes and biomolecules, Table S3: PLS-DA parameters for discrimination of LG and HG BC cytology from normal urothelial cells.
