# Supplementary material for: FTIR Spectroscopic Imaging Supports Urine Cytology for Classification of Low- and High-Grade Bladder Carcinoma
Source: Cancers (Basel). 2021 Nov 16;13(22):5734. doi: 10.3390/cancers13225734 (PMC8616357; doi:10.3390/cancers13225734)
Supplement: Supplementary file 1 [file cancers-13-05734-s001.zip › cancers-1439569-supplementary.pdf]

Supplementary Materials

FTIR Spectroscopic Imaging Supports Urine Cytology for Classification of Low- and High-Grade Bladder Carcinoma

Monika Kujdowicz [1,2], Brygida Mech [2], Karolina Chrabaszcz [2,3], Piotr Chlosta [4], Krzysztof Okon [1,*] and Kamilla Malek [2,*]

[1]Department of Pathomorphology, Faculty of Medicine, Jagiellonian University Medical College, Grzegorzecka 16, 31-531 Krakow, Poland; monika.kujdowicz@uj.edu.pl
[2]Faculty of Chemistry, Jagiellonian University in Krakow, Gronostajowa 2, 30-387 Krakow, Poland; brygida.mech@student.uj.edu.pl (B.M.); karolina.chrabaszcz@ifj.edu.pl (K.C.)
[3]Department of Experimental Physics of Complex Systems, Institute of Nuclear Physics, Polish Academy of Sciences, Radzikowskiego 152, 31-342 Krakow, Poland
[4]Department of Urology, Faculty of Medicine, Jagiellonian University Medical College, Jakubowskiego 2, 30-688 Krakow, Poland; piotr.chlosta@uj.edu.pl

Table S1. Normal, LG and HG BC cell features according to the Paris system.

Cell features	Normal	LG BC	HG BC
**Nuclear to cytoplasmic ratio (N/C)**	<0,5	>0.5	>0,7
**Nuclear hyperchromasia**	-	++	+++
**Irregular nuclear membrane (chromatinic rim or nuclear contour)**	-	++	+++
**Irregular, coarse, clumped chromatin**	-	++	+++
**Pleomorphism**	-	++	+++
**Variation in size and shapes**	-	++	+++
**Scant, pale or dense cytoplasm**	-	+	+++
**Prominent nucleoli**	-	++	+++
**Mitoses**	-	++	+++
**Necrotic debris**	-	++	+++
**Inflammation**	-	+	+++
**Cytoplasmic homogeneity**	+++	+	-

BC – Bladder urothelial carcinoma; LG – low grade; HG – high grade, presence of the feature: + - low, ++ - moderate, +++ - severe.

Table S2. Positions of IR bands observed in FTIR spectra of urine sediment with their assignment to vibrational modes and biomolecules [11,20-25].

Band [cm^{-1}]	Assignment
**994**	RNA and glycogen
**1024**	Carbohydrates and glycolipids (i.e. glycogen and sphingomyelin)
**1052**	DNA, polysaccharides, cholesterol, glycoproteins, glycolipids; ν(C–O-O-C)
**1080**	DNA, RNA and other phosphate containing molecules, glycogen; ν(PO^{2-}), ν(CC)
**1112**	Polysaccharides, ribose, lactate; ν(C-O), ν(CC-OC)
**1153**	Carbohydrates (predominantly glycogen); ν_{as} (CO-O-C)
**1240**	Nucleic acids, phospholipids, phosphoproteins; ν_{as} (PO_2^-)
**1398**	Lipids, free amino acids; ν_{as} (COO^-)
**1455**	Proteins, lipids, carbohydrates, lipopolysaccharides, phospholipids; δ(CH_2, CH_3)
**1516**	Proteins; tyrosine ring ν(CC)
**1546**	Amid II and lipids
**1584**	Lipids, proteins, nucleic acids, lipopolysaccharides; ν_{as} (COO^-), ν(C=N, C=C)
**1623**	Proteins (parallel β-turns) ; C=O, N-H
**1652**	Amide I of α-helix; C=O, N-H
**1718**	Fatty acids and nucleic acids: T-A, G-C (Hoogsteen third strand binding); ν (C=O)
**1740**	Ester group in triglycerides, cholesterol and phospholipids, lipopolysaccharides; ν_s (C=O)
**2850**	Lipids and proteins; ν_{as} (-CH_2)
**2874**	Phospholipids, esters
**2923**	Lipids and proteins; ν_{as} (-CH_2)
**2960**	Lipids and proteins; ν_{as} (-CH_3)

ν – stretching mode, as – antisymmetric, s - symmetric; δ – in-plane deformations (bending); γ – out-of-plane deformations, τ – twisting, (+) – intense signal from band (of whole cells), (+/-) – average intense signal of band, (-/+) – weak visible band, (-) – not observed.

Table S3. PLS-DA parameters for discrimination of LG and HG BC cytology from normal urothelial cells.

**Classification**	**N vs LG**	**N vs HG**
**PLS parameters**	**RMSE / R^2**	**RMSE / R^2**
Calibration	0.55 / 0.70	0.43 / 0.82
Validation	0.59 / 0.65	0.47 / 0.78
Prediction	0.55 / 0.70	0.47 / 0.78

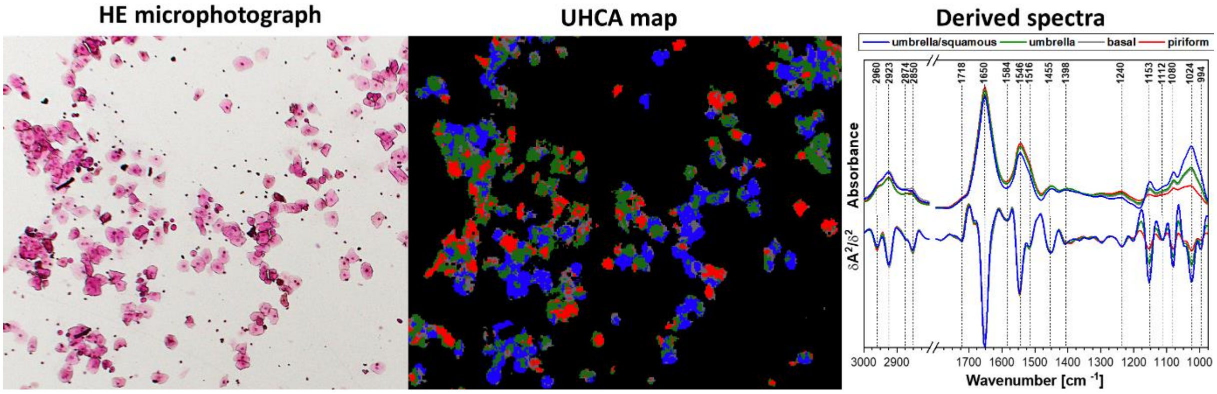

Figure S1. The comparison of HE staining and UHCA-discriminated cells of normal urothelial cells showing their assignment; on right, mean FTIR spectra of UHCA classes (the colors of the spectra correspond to the colors of classes in the UHCA map).

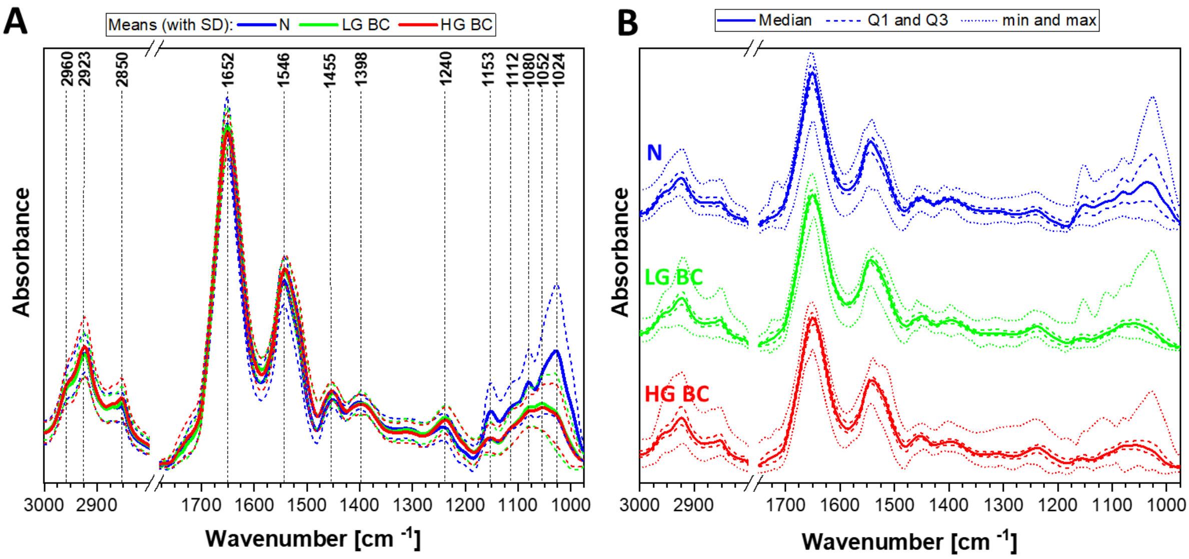

Figure S2. A. Averaged absorbance FTIR spectra (with SD marked as dashed lines). **B.** Median, 1st and 3rd quantile and min and max plots. All spectra were preprocessed after baseline correction and vector normalization.

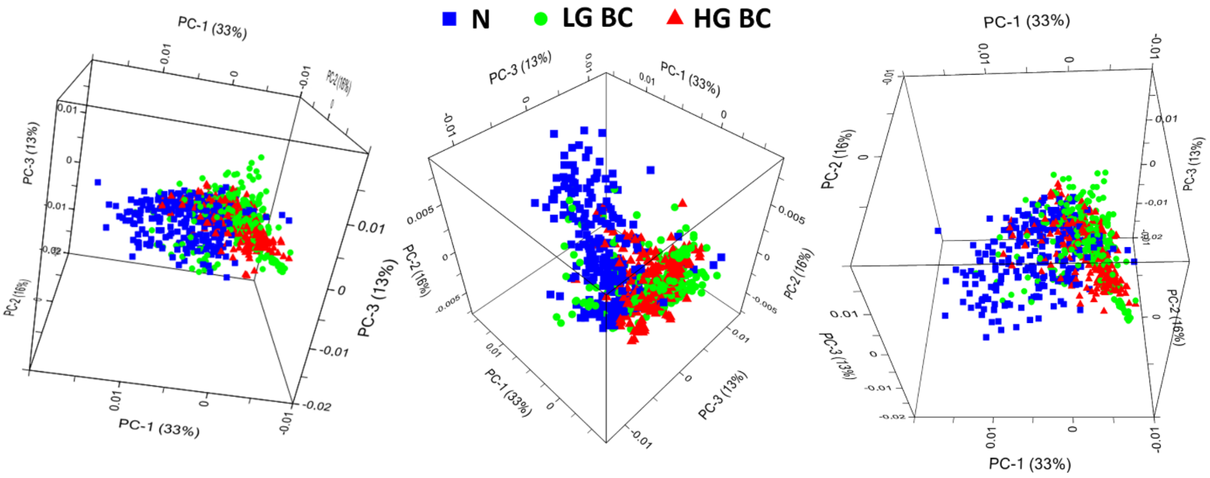

Figure S3. 3D PCA scores plots from Fig. 2B showed in different projections.

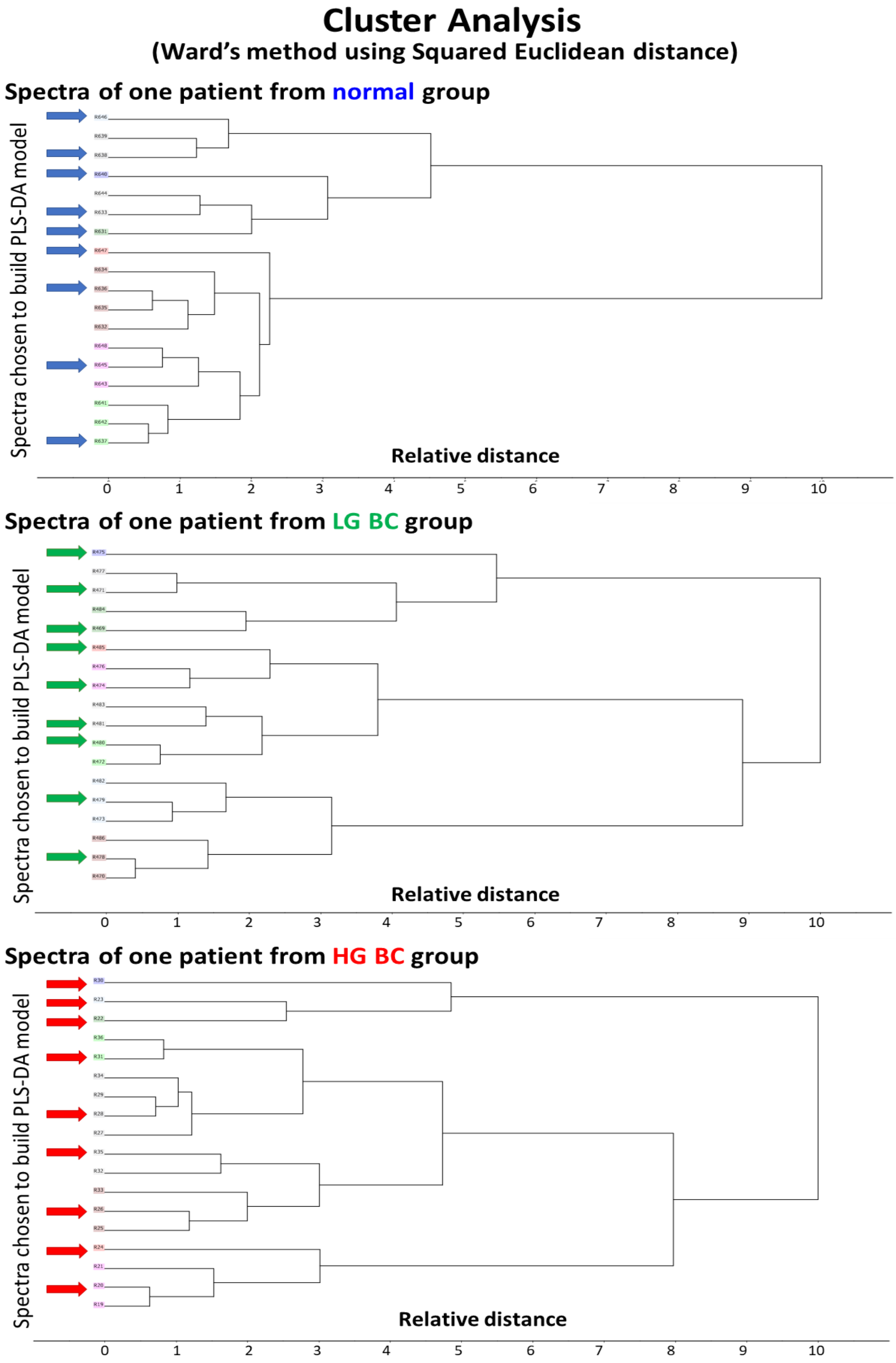

Figure S4. Example of cluster analysis from one of N, LG and HG BC patients. Spectra from the most different branches of Cluster Analysis were chosen to build the model, were marked with arrows and they represent 9 out of 18 spectra from the patient.
